# Biodegradable Mg-Cu alloys with enhanced osteogenesis, angiogenesis, and long-lasting antibacterial effects

**DOI:** 10.1038/srep27374

**Published:** 2016-06-07

**Authors:** Chen Liu, Xuekun Fu, Haobo Pan, Peng Wan, Lei Wang, Lili Tan, Kehong Wang, Ying Zhao, Ke Yang, Paul K. Chu

**Affiliations:** 1 Institute of Metal Research, Chinese Academy of Sciences, Shenyang, China; 2 Department of Materials Science and Engineering, Nanjing University of Science and Technology, Nanjing, China; 3 Shenzhen Institutes of Advanced Technology, Chinese Academy of Sciences, Shenzhen, China; 4 Department of Physics and Materials Science, City University of Hong Kong, Tat Chee Avenue, Hong Kong, China

## Abstract

A series of biodegradable Mg-Cu alloys is designed to induce osteogenesis, stimulate angiogenesis, and provide long-lasting antibacterial performance at the same time. The Mg-Cu alloys with precipitated Mg_2_Cu intermetallic phases exhibit accelerated degradation in the physiological environment due to galvanic corrosion and the alkaline environment combined with Cu release endows the Mg-Cu alloys with prolonged antibacterial effects. In addition to no cytotoxicity towards HUVECs and MC3T3-E1 cells, the Mg-Cu alloys, particularly Mg-0.03Cu, enhance the cell viability, alkaline phosphatase activity, matrix mineralization, collagen secretion, osteogenesis-related gene and protein expressions of MC3T3-E1 cells, cell proliferation, migration, endothelial tubule forming, angiogenesis-related gene, and protein expressions of HUVECs compared to pure Mg. The favorable osteogenesis and angiogenesis are believed to arise from the release of bioactive Mg and Cu ions into the biological environment and the biodegradable Mg-Cu alloys with osteogenesis, angiogenesis, and long-term antibacterial ability are very promising in orthopedic applications.

Degradable magnesium-based metals have been widely investigated both *in vitro* and *in vivo*^1^,^2^,^3^ due to their unique degradation property in physiological environment, which effectively avoids the repeated surgery for implant removal and decreases the costs and risks to patients, offering a practical and promising alternative for bone regeneration and other orthopedic applications^4^,^5^,^6^. Besides having similar mechanical properties as human bones^7^, magnesium alloys have positive effects on growth of bone tissues growth. The products arising from natural degradation of magnesium influences proliferation and apoptosis of osteoblasts and osteoclasts^8^ and a high magnesium ion concentration has been observed to introduce bone cell activation^9^,^10^. Some magnesium alloys such as Mg-Sr alloys containing trace metallic elements such as strontium may also stimulate new bone formation and increase new bone quality^11^,^12^. In addition to promoting osteogenesis, magnesium alloys can inhibit bacteria-induced infection after implantation into bones^13^ thereby effectively decreasing morbidity and mortality. In the fight against infection, surgical procedures are being improved but with diminishing return^14^,^15^,^16^,^17^. It has been observed that an alkaline environment with a high pH value produced by degradation of magnesium may reduce the bacterial activity^18^,^19^. However, as most aquatic organisms live in a neutral pH range between 6.5 and 8 and the pH in the human body is regulated and stabilized by a buffering system consisting of weak acid-conjugate base pairs in order to ensure optimal physiological performance of enzymes and other biomolecules^20^,^21^. Therefore the high alkalinity caused by Mg degradation is buffered *in vivo* if the time is long enough^22^ and so antibacterial effects arising from alkalinity may only work in the early stage of implantation. Murdoch *et al.* observed implant-associated infection in 15 out of 44 patients with long-term implantation of prosthetic joints. Infection occurs not only in the early period after implantation but also during the entire lifetime of the implant^23^,^24^. Hence, a long-lasting antibacterial effect throughout the entire implantation period is necessary for Mg implants *in vivo*.

Copper (Cu) is one of the effective antibacterial metals^25^,^26^ and incorporation of Cu into stainless steels^25^,^27^,^28^, titanium alloys^29^ and cobalt-based alloys^30^ has been observed to enhance the resistance against bacteria such as *Staphylococcus aureus* (*S. aureus*) and *Escherichia coli* (*E. coli*). Cu is also a vital trace element playing an important role in the immune system^31^,^32^, restoring the normal rate of bone resorption in bone metabolism^33^, and enhancing collagen fiber deposition and so is attractive in the field of bone engineering^34^. In fact, both osteoinduction and osteoclast activities are affected by Cu deficiency^35^ and Cu stimulates endothelial cell proliferation and enhances angiogenesis^36^,^37^ which is a crucial physiological event in bone regeneration^38^. For instance, rabbits fed with a Cu-deficient diet were unable to provide the normal angiogenic response^39^.

It is usual that 4∼16 weeks of degradation are thought to be appropriate for Mg-based implants in human body to ensure fracture stabilization and healing^40^. Even though from the material perspective, Cu alloying tends to accelerate the Mg degradation, the degradation rate can be adjusted by tailoring Cu content in the alloys to meet the clinic demands. Cu alloying also tends to diminish mechanical properties of magnesium alloys but in orthopedic applications, such as in bone defect repair and osteomyelitis treatment, natural degradation may be beneficial and so Mg-Cu alloys may be more suitable. However, there has been very little research on Cu-containing magnesium alloys and the related biological performance. In this study, a series of novel Mg-Cu alloys, namely Mg-0.05Cu, Mg-0.2Cu, and Mg-0.5Cu, is developed. The biological properties including osteogenesis and angiogenesis performance as well as antibacterial property are investigated comprehensively *in vitro* to offer insights into the feasibility of orthopedic application. Additionally, the underlying mechanisms promoting osteogenesis, angiogenesis, and bacterial resistance are analyzed and discussed.

## Results

### Microstructure

**Figure 1:**
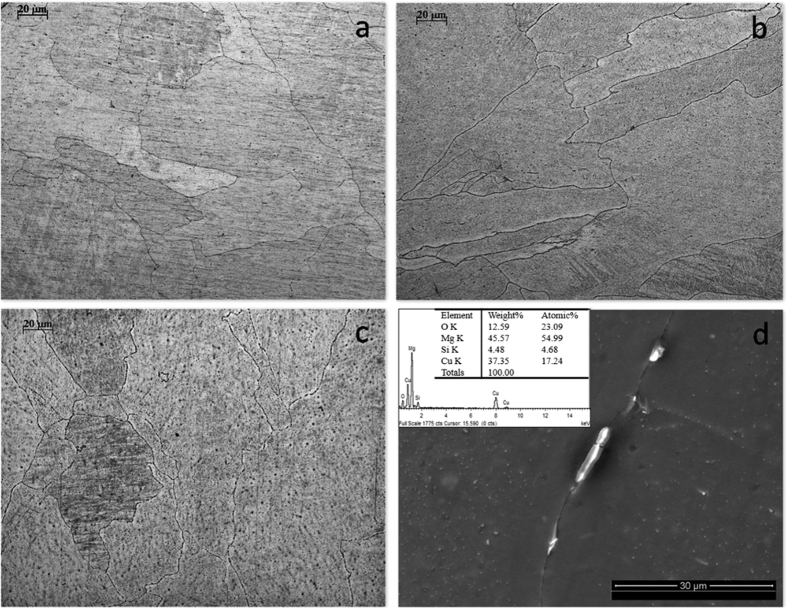
Microstructures of (**a**) as-cast Mg-0.03Cu, (**b**) Mg-0.19Cu, and (**c**) Mg-0.57Cu; (**d**) Morphology and EDS results of the second phase.

Figure 1(a–c) display the optical microstructures of the as-cast Mg-Cu alloys revealing the matrix and granular second phases. The average grain size is about 100 μm and there is no observable difference among the various Mg-Cu alloys. As shown in Fig. 1(a,b), a small amount of second-phase precipitate shown as black spots exists in the grains. With increasing Cu content, more second-phase particles are observed in the grains and distributed discontinuously along the grain boundaries (Fig. 1c). According to EDS (Fig. 1d) and the Mg-Cu binary phase diagram, the Mg_2_Cu intermetallic is the only precipitated phase in the alloys.

### Mechanical properties

**Figure 2:**
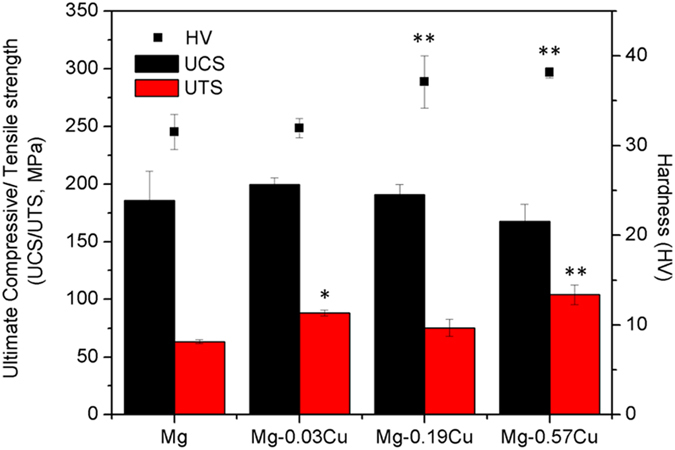
Vicker’s hardness (HV), ultimate compressive strength (UCS), and ultimate tensile strength (UTS) of the as-cast Mg-Cu alloys. ^*^p < 0.05 and ^**^p < 0.01 compared to pure Mg.

Figure 2 shows the Vicker’s hardness (HV), ultimate compressive strength (UCS), and ultimate tensile strength (UTS) of the as-cast Mg-Cu alloys. The HV of the Mg-0.03Cu (31.94) is slight larger than that of pure Mg (31.53) but smaller than those of Mg-0.19Cu (37.10) and Mg-0.57Cu (38.12). Similarly, the largest UTS is obtained from the Mg-0.57Cu alloy (104.00 MPa) and it nearly double that of pure Mg (63.33 MPa). This can be explained by the increase in the Mg_2_Cu phase along the grain boundary and resulting pinning mechanism. Different from HV and UTS, the UCS of the Mg-0.03Cu (199.67 MPa) is the largest among the Mg-Cu alloys and is larger than that of pure Mg (185.67 MPa). In summary the as-cast Mg-Cu alloys possess better mechanical properties than pure magnesium.

### *In vitro* immersion

**Figure 3:**
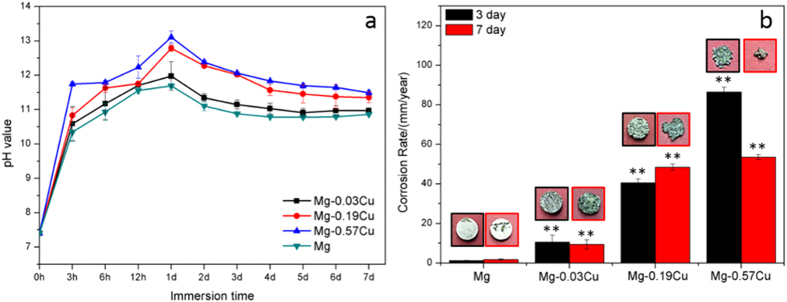
(**a**) pH change during immersion of the Mg-Cu alloys and pure Mg in Hank’s solutions and (**b**) Corrosion rates after 3 days and 7 days. ^**^p < 0.01 compared to pure Mg.

Figure 3(a) presents the pH variation during immersion of the Mg-Cu alloys and pure Mg in Hank’s solution. There is a gradual rising trend in the initial stage reaching a peak after 1 day due to OH^−^ 41. As corrosion proceeds, more degradation products cover the samples to retard the corrosion rate and the pH becomes stable. Nonetheless, as shown in Fig. 3(a), the pH of the Mg-Cu alloys is always higher than that of pure Mg during the immersion period. In particular, Mg-0.57Cu has the largest corrosion rate, indicating that more Cu results in more severe galvanic corrosion in the Mg_2_Cu/Mg system.

Figure 3(b) shows the corrosion rates as well as the images of the Mg-Cu alloys and pure Mg after immersion for 3 and 7 days. Compared to pure Mg, the corrosion rates of the Mg-Cu alloys increase significantly after 3 and 7 days with Mg-0.57Cu having the largest Cu concentration exhibiting the most severe corrosion. After immersion for 3 days, many corrosion pits are present as opposed to the almost intact pure Mg surface. After 7 days, both the Mg-Cu alloys and pure Mg exhibit pitting corrosion and erosion on the Mg-Cu alloys continues to be more substantial, indicating that Cu alloying accelerates Mg corrosion.

**Figure 4:**
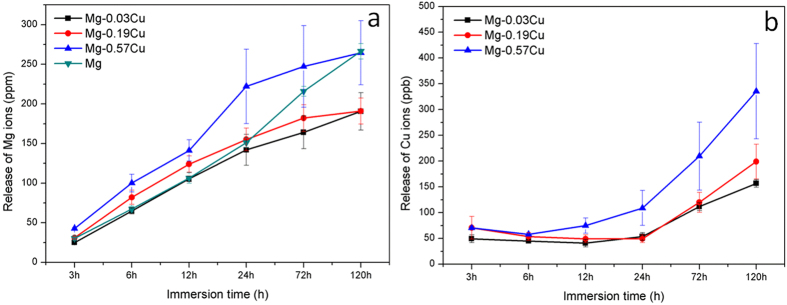
(**a**) Mg and (**b**) Cu release from the Mg-Cu alloys after different immersion periods in Hank’s solutions.

Figure 4 shows the degree of Mg and Cu release from the Mg-Cu alloys after immersion for different time in Hank’s solution. As shown in Fig. 4(a), the amount of Mg leached from the Mg-Cu alloys and pure Mg increase almost linearly with time with Mg-0.57Cu showing the largest Mg release. During the first 24 h, the rate of release rate from pure Mg is close to that of Mg-0.03Cu alloy and considerably less than those of Mg-0.19Cu and Mg-0.57Cu. Subsequently, the release rates of Mg-0.03Cu and Mg-0.19Cu alloys are at a lower level than pure Mg due to the formation of protective layer composed of corrosion products. These results are consistent with the pH variation shown in Fig. 3(a). Figure 4(b) shows the highest Cu release from Mg-0.57Cu followed by Mg-0.19Cu and Mg-0.03Cu. The Cu concentration varies slowly in the early stage but increases rapidly after 24 h.

### Antibacterial properties

**Figure 5:**
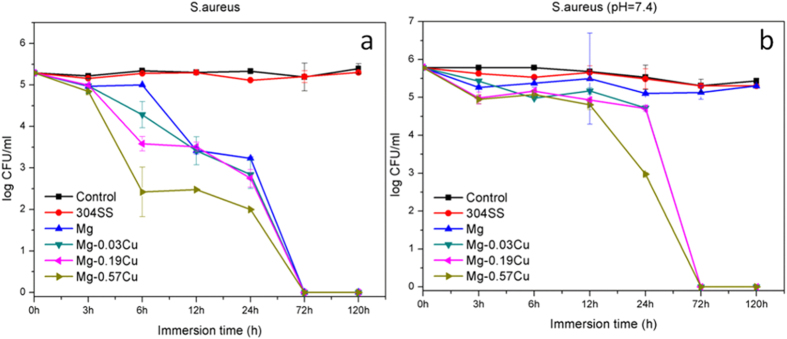
Colony-forming unit/mL of *S. aureus* after incubation with extractions of different samples in Hank’s solution for various intervals at (**a**) normal pH values and (**b**) neutral pH of 7.4.

Figure 5(a) shows the colony-forming unit (CFU)/mL of *S. aureus* after incubation in the extracts for various time intervals. The *S. aureus* colonies on the Mg-Cu alloys and pure Mg extracts decrease with immersion time whereas those of the control extract of 304 stainless steel are the same as that of the blank control disclosing that the control group has no antibacterial effects. The *S. aureus* colony counts of the Mg-Cu alloy groups are always less than those of pure Mg group and the effects are more pronounced for a larger Cu concentration. After 72 h, the colony counts of the Mg-Cu alloys and pure Mg groups are all zero.

The antibacterial effect of Cu released from the Mg-Cu alloys against *S. aureus* in the neutral environment is shown in Fig. 5(b). The antibacterial performance is not significant initially. After 72 h, the colony-forming units on the Mg-0.19Cu and Mg-0.57Cu are almost zero (Fig. 5b). In comparison, pure Mg shows no antibacterial effect in the neutral environment. The colony count of Mg-0.57Cu diminishes abruptly after 24 h and is obviously less than those of Mg-0.19Cu and Mg-0.03Cu. It is believed that more Cu release improves the antibacterial ability of the Mg-Cu alloys indicating the role played by Cu besides the high pH. All in all, the Mg-Cu alloys show stronger antibacterial effect than pure Mg, especially in the early period (6 h) due to both the alkaline environment (Fig. 3a) and continuous leaching of Cu (Fig. 4b) as a result of natural degradation.

### Cytotoxicity

**Figure 6:**
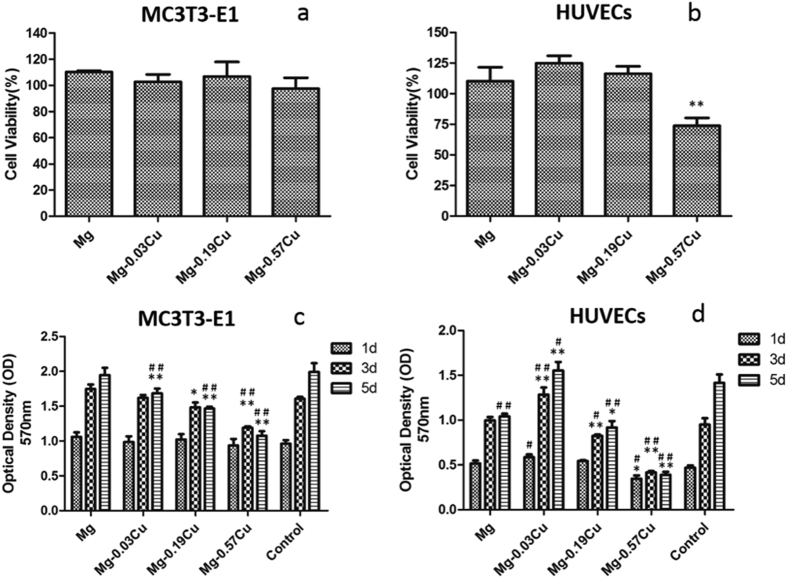
Cell viability after incubation for 24 h: (**a**) MC3T3-E1 cells and (**b**) HUVECs; OD values of Mg-Cu alloys and pure Mg extraction media after incubation for 1, 3, and 5 days: (**c**) MC3T3-E1 cells, (**d**) HUVECs. ^*^p < 0.05 and ^**^p < 0.01 compared to pure Mg; ^#^p < 0.05 and ^##^p < 0.01compared to the control.

The viability of MC3T3-E1 and HUVECs cells after incubation for 24 h for the Mg-Cu alloys and pure Mg extraction media is presented in Fig. 6(a,b), respectively. The pre-osteoblast viability of both Mg-0.03Cu and Mg-0.19Cu is larger than 100%, whereas that of Mg-0.57Cu is slightly smaller than that of pure Mg and other alloys. The Mg-Cu alloys exhibit a descending HUVECs viability from 125 to 75% with increasing Cu concentrations in the Mg-Cu alloys and the HUVECs viability of pure Mg is between that of Mg-0.19Cu and Mg-0.57Cu. The results furnish evidence that the Mg-Cu alloys are not toxic according to the ISO 10993-5:2009 and in fact, Mg-0.03Cu and Mg-0.19Cu even stimulate the growth of MC3T3-E1 and HUVECs cells.

### Cell proliferation

Proliferation of the MC3T3-E1 and HUVECs cells in the extraction media of Mg-Cu alloys and pure Mg for 1, 3, and 5 days are depicted in Fig. 6(c,d). With regard to the MC3T3-E1 cells, there is no significant difference in the cell number among the Mg-Cu alloys, pure Mg, and control groups on day 1. However, after 3 and 5 days, less cell proliferation is observed from the Mg-Cu alloys. Cu appears to depresses MC3T3-E1 cell proliferation and in the case of HUVECs, Mg-0.03Cu shows the most cell proliferation than other samples after 1, 3, and 5 days while Mg-0.57Cu exhibits the opposite response. Generally, a lower Cu concentration such as that in Mg-0.03Cu leads to a more cell proliferation. When the Cu content is 0.57 wt%, negative cell growth is in fact observed.

### Cell adhesion

**Figure 7:**
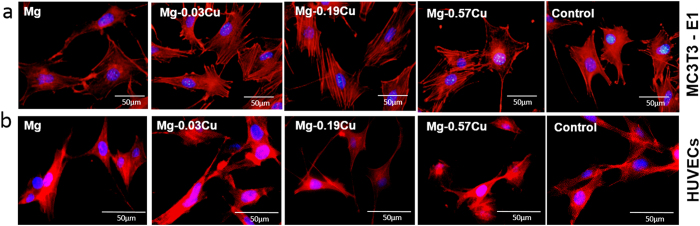
Adhesion of MC3T3-E1 cells and HUVECs in different extracts. Cytoskeleton staining of (**a**) MC3T3-E1 cells and (**b**) HUVECs after incubation for 12 h in different extracts with DAPI for nuclei (blue) and rhodamine phalloidin for F-actin stress fibers (red).

Figure 7(a) exhibits the cytoskeletons of the MC3T3-E1 cells after immersion for 12 h in different extracts. Compared to the control, the attached cells for the pure Mg and Mg-Cu alloys extracts show more spreading and superior filopodia extension. The MC3T3-E1 cells cultured in the Mg-0.03Cu and Mg-0.19Cu extracts show more plump focal adhesion *via* well-organized F-actin stress fibers (red filaments) compared to pure Mg and Mg-0.57Cu. Similar morphologies are revealed in Fig. 7(b). As the Cu concentration increases, aggregation of HUVECs diminishes and the cell status deteriorates slightly, indicate that the Mg-0.03Cu and Mg-0.19Cu extracts are favorable to initial attachment and spreading of HUVECs and MC3T3-E1 cells.

### Osteogenic differentiation

**Figure 8:**
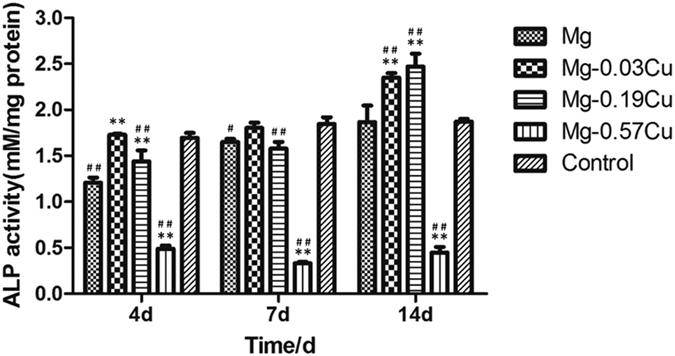
ALP activity of MC3T3-E1 cells after incubation for 4, 7, and 14 days in different extracts. ^**^p < 0.01 compared to pure Mg; ^#^p < 0.05 and ^##^p < 0.01compared to the control.

Figure 8 shows the ALP activity of the MC3T3-E1 cells after incubation for 4, 7, and 14 days in different extracts. The ALP activity of Mg-0.03Cu is higher than that of pure Mg throughout the experimental period. Mg-0.19Cu shows slightly lower ALP activity than Mg-0.03Cu on both days 4 and 7 and the highest ALP activity is observed from Mg-0.19Cu group on day 14. The ALP activity of Mg-0.57Cu is lower than that of other samples.

**Figure 9:**
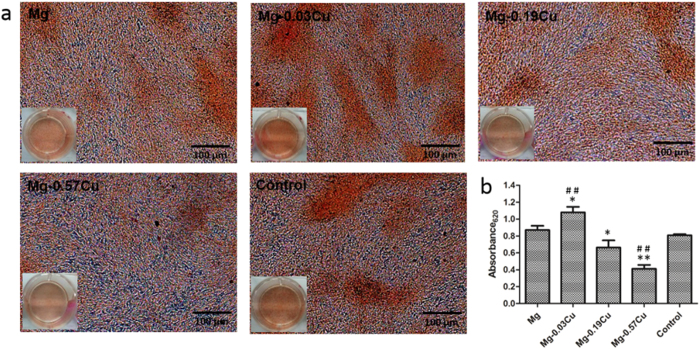
(**a**) Extracellular matrix mineralization of MC3T3-E1 cells after incubation for 15 days in different extracts and (**b**) colorimetrically quantitative analysis (**b**). *p < 0.05 and **p < 0.01 compared to pure Mg; ^##^p < 0.01compared to the control.

**Figure 10:**
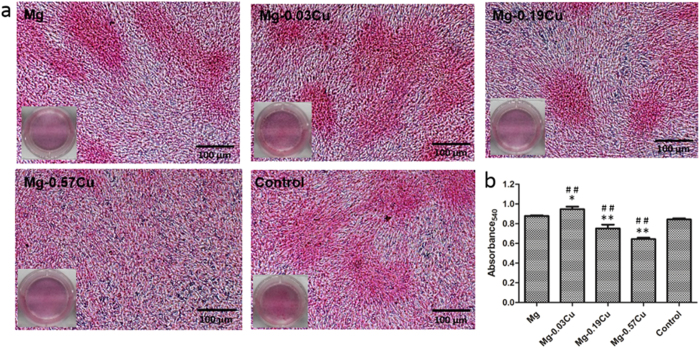
(**a**) Collagen secretion from MC3T3-E1 cells after incubation for 15 days in different extracts and (**b**) colorimetrically quantitative analysis. *p < 0.05 and **p < 0.01 compared to pure Mg; ^##^p < 0.01compared to the control.

Figure 9 displays the ECM mineralization of MC3T3-E1 cells after incubation for 15 days in different extracts together with the corresponding quantitative colorimetric analysis. Figure 9(a) reveals mineralized calcium nodules on all samples and Fig. 9(b) shows more ECM mineralization on Mg-0.03Cu than pure Mg and the control. The ECM mineralization levels on Mg-0.19Cu and Mg-0.57Cu are less than those on the pure Mg and control. Particularly, Mg-0.57Cu shows significantly depressed ECM mineralization. Figure 10 depicts collagen secretion from MC3T3-E1 cells after incubation for 15 days in different extracts together with the corresponding quantitative colorimetric analysis. Figure 10(a) shows that denser collagen is secreted in the Mg-0.03Cu extract than the pure Mg and control. The collagen stained in the extract of Mg-0.19Cu is less than that of Mg-0.03Cu and no significant clumps of collagen is deposited in the Mg-0.57Cu extract. According to the quantitative analysis in Fig. 10(b), collagen secretion in the Mg-0.19Cu and Mg-0.57Cu extracts diminishes by about 86% and 73% compared to pure Mg and 89% and 76% relative to the control. Collagen secretion of Mg-0.03Cu group is promoted to about 108% and 112% compared to the pure Mg and control, respectively.

### Osteogenesis-related gene and protein expressions

**Figure 11:**
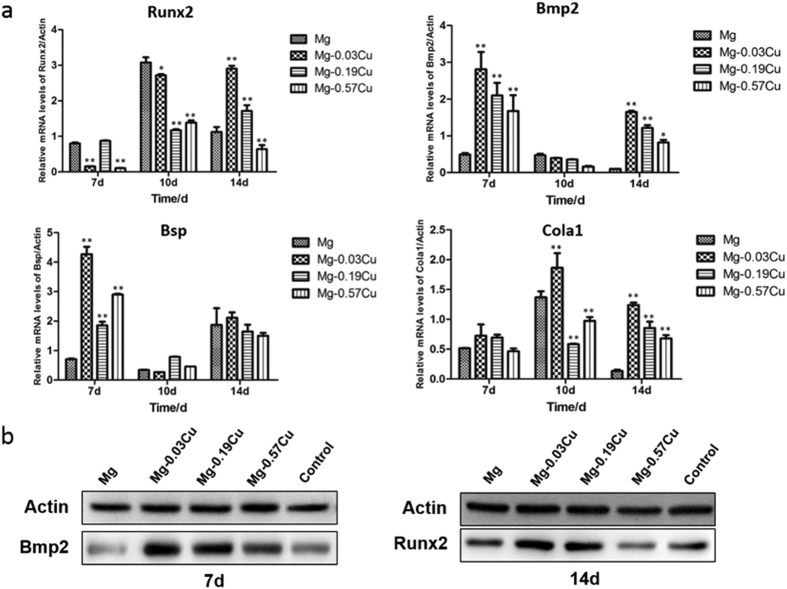
(**a**) Osteogenesis-related gene expressions by MC3T3-E1 cells after incubation for 7, 10 and 14 days in different extracts and (**b**) osteogenesis-related protein expressions by MC3T3-E1 cells at the time point showing high gene expressions in different extracts. *p < 0.05 and **p < 0.01 compared to pure Mg.

Figure 11(a) shows the osteogenesis-related gene expressions of Runx2, Bmp2, Bsp, and Cola1 after incubation for 7, 10, and 14 days with MC3T3-E1 cells in different extracts. In general, the gene expressions are time dependent. After 7 days, higher (p < 0.01) Bmp2 and Bsp expressions are found from the Mg-Cu extracts than pure Mg and the Bmp2 and Bsp expressions of Mg-0.03Cu are the highest being 5.7 and 6.1 times of those of pure Mg. With regard to the gene Runx2, the expression of Mg-0.19Cu is similar to that of pure Mg, but those of Mg-0.03Cu and Mg-0.57Cu groups are depressed (p < 0.01). The Cola1 expressions of all the samples show no significant difference. After culturing for 10 days, the Cola1 expression Mg-0.03Cu is higher (p < 0.01) and that of Runx2 is slightly lower than that of pure Mg. There is no statistical difference in the Bmp2 and Bsp expressions among the various groups. In addition, the gene expression trend is similar on day 14. The Runx2, Bmp2, Bsp, and Cola1 expressions of MC3T3-E1 cells of all the Mg-Cu samples decline gradually with Cu concentration but those of the Mg-Cu samples are consistently higher than that of pure Mg. Mg-0.03Cu shows the most significant Runx2, Bmp2, Bsp, and Cola1 expressions of MC3T3-E1 cells followed by Mg-0.19Cu and Mg-0.57Cu.

To investigate osteogenesis on the protein level, osteogenesis-related protein expressions are measured by western blots. As shown in Fig. 11(b), the Bmp2 protein is expressed strongly by Mg-0.03Cu and Mg-0.19Cu after 7 days. A high expression of the Runx2 protein is observed from Mg-0.03Cu after 14 days whereas the Runx2 expression is down-regulated with increasing Cu contents. The effects of Cu on the osteogenesis-related protein expression and gene expression are consistent.

### Cell migration

**Figure 12:**
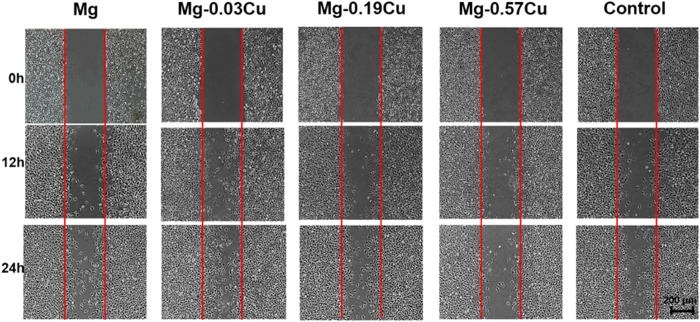
*In situ* directional migration of HUVECs in different extracts based on the wound-healing assay after incubation for more than 24 h.

Figure 12 displays the *in situ* directional migration of HUVECs in different extracts after incubation for more than 24 h by means of the wound-healing assay. The HUVECs migrate gradually into the free gaps along with time. Mg-0.03Cu shows more cell migration toward the wound than pure Mg and Mg-0.19Cu. The wound of Mg-0.03Cu is nearly covered with cells after 24 h. The cell migration rate decreases with Cu concentration and cells migration in the gap of Mg-0.57Cu does not increase much with time. Among the various samples, Mg-0.03Cu shows the best cell migration.

### *In vitro* angiogenesis

**Figure 13:**
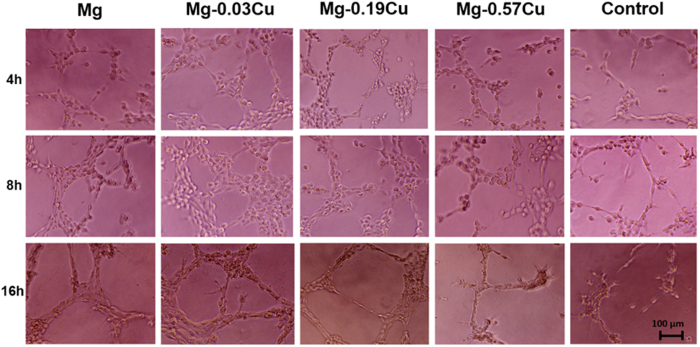
*In vitro* angiogenesis of HUVECs cultured on the Matrigel™ basement membrane matrix in the presence of different extracts for 4, 8, and 16 h.

Figure 13 shows the *in vitro* angiogenesis of HUVECs cultured on the Matrigel™ basement membrane matrix for 4, 8, and 16 h. After 4 h, the HUVECs in the control group are stretched forming short lines and branch nodes (node) typical of the primary stage of angiogenesis^42^. The HUVECs of the Mg-Cu alloy and pure Mg groups show more nodes and they also connect to each other forming a more complex morphology of angiogenesis such as mesh-like circles than the cells cultured on the control. Moreover, the HUVECs on Mg-0.03Cu show more close circles than other samples. After 8 h, formation of circles on the Mg-Cu alloys and pure Mg is accelerated compared to the control. More parallel cell lines (tubers) indicative of the late phase of angiogenesis are observed, especially Mg-0.03Cu. After 16 h, the HUVECs begin to undergo apoptosis due to the limited nutrition supply. At this point, few cells can be observed from the control group. The cells on Mg-0.57Cu show apoptosis and the cell number decreases sharply. In contrast, the HUVECs on Mg-0.03Cu, Mg-0.19Cu, and pure Mg groups do not show obvious apoptosis. Many nodes, circles, and tubes are still evident, especially Mg-0.03Cu, although the cell number is less than that after 8 h. The results suggest that the Mg-Cu alloys with a small Cu concentration stimulates angiogenesis.

### Aortic ring model of angiogenesis

**Figure 14:**
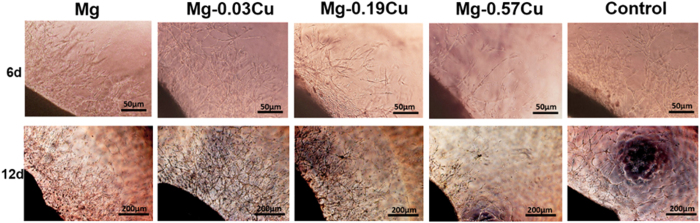
Microvessel outgrowth from the edges of the SD rat thoracic aortic rings embedded in rat tail collagen gels and cultured in different extracts after incubation for 6 and 12 days.

Figure 14 demonstrates the microvessel outgrowth from the edges of the SD rat thoracic aortic rings embedded in rat tail collagen gels after 6 and 12 days. After 6 days, the thoracic aortic rings in all samples show a complex array of microvessels arising from the severed edges of the rings and sprouting into the collagen gels. The number of the microvessles in Mg-0.03Cu is similar to that of the control. As the Cu concentration increases, adhesion and spreading of the endothelial cells are adversely affected suggesting diminished angiogenic response. After 12 days, more branching of the microvessels is observed migrating into the gels, especially Mg-0.03Cu. In addition, the obvious mesh-like endothelial nature is observed from Mg-0.03Cu, Mg-0.19Cu, and the control, but for pure Mg, the endothelial cells do not connect with each other to form a network although more cells are produced during the process, indicating relatively weak angiogenesis.

### Angiogenesis-related gene and protein expressions

**Figure 15:**
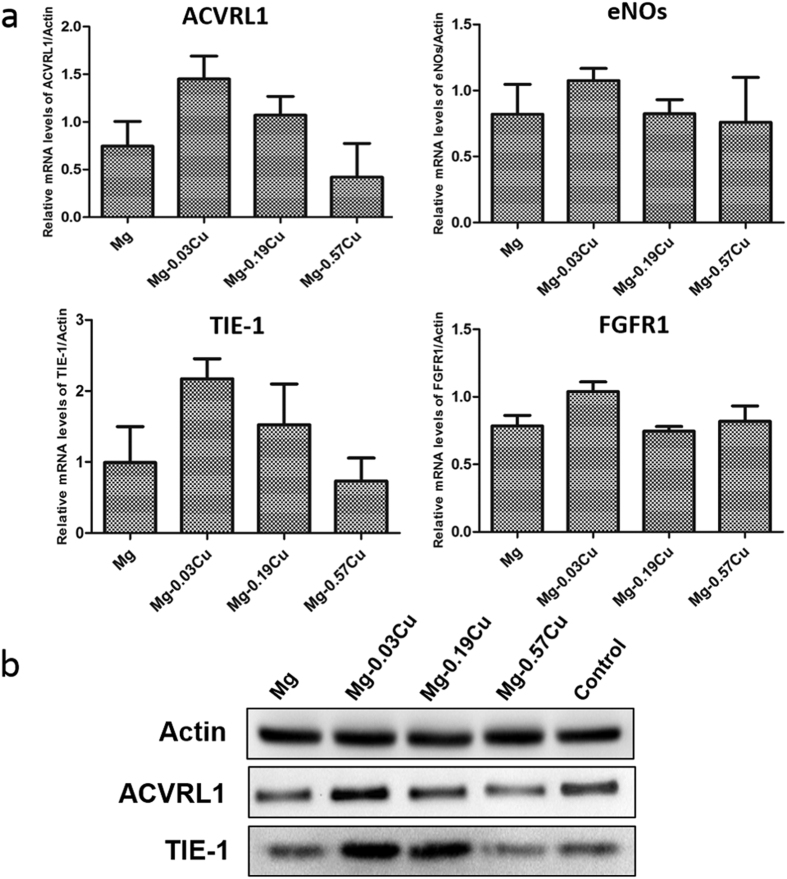
(**a**) Angiogenesis-related gene expressions and (**b**) typical protein expressions by HUVECs after incubation for 3 days in different extracts.

Figure 15(a) shows the angiogenesis-related gene expressions of ACVRL1, eNOs, TIE-1, and FGFR1 after HUVECs incubation in different extracts for 3 days by means of real-time PCR examination. In general, Mg-0.03Cu exhibits the highest gene expression followed by Mg-0.19Cu. Mg-0.57Cu group shows the least at every time point. The ACVRL1 and TIE-1 expression of the Mg-0.03Cu group is most up-regulated and almost twice that of pure Mg. The ACVRL1 and TIE-1 expressions are down-regulated with increasing Cu concentration and a similar diminishing tendency is observed from the eNOs and FGFR1 expressions, whereas that of Mg-0.19Cu and Mg-0.57Cu has the same level as pure Mg. Mg-0.03Cu shows the most noticeable auxo-action on the ACVRL1, eNOs, TIE-1 and FGFR1 expressions. The intracellular protein expressions of ACVRL1 and TIE-1 after 3 days is further analyzed by western blot. As shown in Fig. 15(b), high-level ACVRL1 and TIE-1 protein expressions are detected from Mg-0.03Cu demonstrating that Mg-0.03Cu can up-regulate the ACVRL1 and TIE-1 expressions of HUVECs and the results are consistent with the gene expressions.

## Discussion

**Figure 16:**
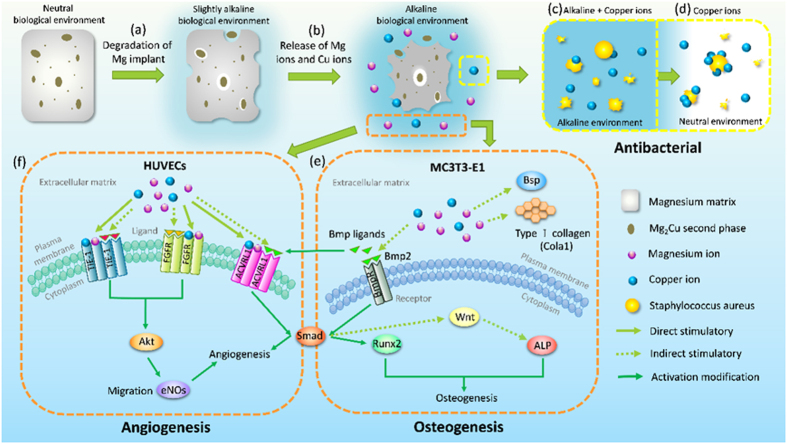
Schematic process illustraing *in vivo* degradation and possible antibacterial, osteogenesis, and angiogenesis mechanism for the Mg-Cu alloys.

The ideal bone repair materials should have the capability to guide proliferation and differentiation of osteoblasts, stimulate angiogenesis of endothelia cells, and resist bacterial infection. Magnesium and its alloys provide an alternative way due to their light weight, natural degradation, load bearing, and stimulatory effects on bone formation^4^. Herein, Mg-Cu alloys that inhibit bacteria growth while stimulating response from in preosteoblasts (MC3T3-E1) and endothelial cells (HUVECs) are designed, fabricated, and investigated systematically. The schematic in Fig. 16 illustrate *in vitro* degradation and the possible antibacterial, osteogenic, and angiogenic mechanisms. In the physiological environment, a galvanic couple is formed due to different corrosion potentials between the Mg_2_Cu phase and Mg matrix. The immersion tests (Figs 3 and 4) show that the corrosion resistance deteriorates with increasing Cu contents. It is because there are more Mg_2_Cu precipitates in the Mg matrix acting as the cathode consequently inducing more serious galvanic corrosion (Fig. 16a). The Cu content is thus an important factor and this study provides insights.

Degradation of Mg-Cu is accompanied by a gradual increase in pH^43^. As bacteria can usually survive in the pH range between 6.0 and 8.0 but an acidic or alkali environment is not conducive to bacteria growth^19^,^44^,^45^. In our study, the high pH (Fig. 3a) caused by degradation of Mg-Cu proves to be detrimental to bacteria (Fig. 16c). However, in the physiological environment, the high alkalinity is buffered in the long term *via* the homeostasis system comprising weak acid-conjugate base buffer pairs^20^,^21^,^22^. Hence, long-term infection may not be avoided. Here, the Mg-Cu alloys deliver better antibacterial performance than pure Mg due to the synergistic effects of high alkalinity (Fig. 3a) and Cu release (Fig. 4b). Figure 5(b) shows that the number of *S. aureus* colonies on the Mg-Cu alloys groups declines with time but the pure Mg has no antibacterial effect at the same neutral pH suggesting the additional role played by Cu release (Fig. 4b). In fact, the larger the degree of Cu release, the more potent is the antibacterial ability. The antibacterial mechanism *in vivo* is expected to have two stages. In the first stage, the antimicrobial effects are rendered by the high alkalinity and Cu leaching (Fig. 16c). As time elapse, the pH is stabilized by the body and the antibacterial ability stems from Cu release as the Mg-Cu alloy degrades naturally (Fig. 16d). The combined processes provide long-lasting antibacterial effects minimizing infection and providing a stable environment for bone growth after surgery.

The maximum amount of Cu released from Mg-0.57Cu is 335 μg/L (ppb) after immersion for 120 h in Hank’s solution (Fig. 4b) equivalent to 268 μg/cm^2^ for an immersion ratio of 1.25 cm^2^/ml. If a Mg-0.57Cu implant with dimensions of Φ10 × 3 mm^3^ in implanted into the body, the Cu release is about 0.67 mg per day which is smaller than the recommended daily intake (RDI) (0.9 mg) and tolerable upper intake level (UL) (10 mg) of Cu for adults^46^. It should be mentioned that the *in vivo* corrosion rate is normally smaller than *in vitro* ones as it has been reported that the corrosion rate *in vivo* is on the average 1–5 times smaller than that *in vitro*^47^. Therefore, the biosafety requirement is satisfied. In order to further confirm the biological safety, a comprehensive evaluation including cytotoxicity, cell proliferation, and adhesion of MC3T3-E1 and HUVECs cells which are typical cell lines pertinent to bone formation process is performed and described. In addition to no cytotoxicity, Mg-0.03Cu and Mg-0.19Cu exhibit good cell adhesion (Fig. 1S) as well as high cell viability and proliferation. The *in vitro* results provide evidence that Mg-0.03Cu and Mg-0.19Cu are highly compatible *in vivo*^48^.

Osteogenic differentiation on the Mg-Cu alloys is assessed and the ALP activities of Mg-0.03Cu and Mg-0.19Cu are high after 14 days incubation and better than those on Mg-0.57Cu and pure Mg control (Fig. 8). As an early marker of osteogenic differentiation, a high level of ALP expression suggests that Mg-0.03Cu and Mg-0.19Cu favor osteoblastic differentiation. ECM mineralization is a marker of late osteogenic differentiation^49^ and type I collagen secretion plays a crucial role in osteogenic differentiation^50^. In this study, Mg-0.03Cu induces ECM mineralized nodule formation and enhances collagen secretion. Consistent results are obtained from the RT-PCR studies and the expressions of nearly all the genes, namely Runx2, Bmp2, Bsp, and Cola1, are up-regulated for Mg-0.03Cu. Mg-0.19Cu underperforms compared to Mg-0.03Cu but is better than the other samples. The osteogenesis-related protein expressions are consistent with the gene expressions further confirm the superior osteogenesis ability of the Mg-Cu alloys. All in all, Mg-0.03Cu with a small Cu concentration possesses the best osteogenesis inducing ability which bodes well for faster bone maturation and implant/bone bonding^51^.

The ALP activity, extracellular matrix deposition, collagen secretion and osteogenesis-related gene, and protein expressions observed from Mg-0.03Cu are enhanced compared to the pure Mg and control. It may be attributed to the release of bioactive ions during degradation. Magnesium plays a vital role in the formation of biological apatite and bone metabolism and Mg deficiency may lead to insufficient bone growth and increased bone resorption^52^,^53^,^54^. At the same time, Cu is an essential elements for physiological processes of bone cells and Cu imbalance can lead to osteoporosis and other diseases. Cu may influence bone metabolism such as bone resorption^33^,^55^. A schematic illustrating the possible osteogenesis mechanism is shown in Fig. 16(e). Under physiological conditions, Mg and Cu ions are released from the Mg-Cu alloys (Fig. 16b). Moderate amounts of Mg and Cu increase the activity of bone morphogenetic protein-2 (Bmp2) that belongs to the transforming growth factor-beta (TGF-β) superfamily and induces bone formation^56^,^57^. The Bmp2 ligand interconnects with the receptor and activates the Smad protein signal, which is one of the most important signaling molecules in the downstream of the bone morphogenetic proteins receptors (BmpR). Subsequently, the activated Smad complexes enter the cell nuclei, identify the DNA, and regulate the expression of the nucleus receptor Runx2^58^. Runx2 is a major target gene shared by TGF-β and Bmp signaling pathways and cooperation between Runx2 and Bmp-activated Smads induces osteoblast-specific gene expression and osteogenesis differentiation^59^. The proper amount of Mg and Cu may indirectly induce the expression of early osteogenic marker (ALP) by Bmp2 through the Wnt pathway, which plays a role in bone growth and is important to the establishment of the peak bone mass^60^. Trace amounts of Mg and Cu may act as co-factors impacting the bone mineralization process with the Bsp and Cola1 in the ECM.

Apart from the osteogenesis and antibacterial performance, the angiogenesis ability is another important issue. It is well known that the bone vasculature plays a pivotal role in bone growth, remodeling, and homeostasis. An active blood vessel network is an essential pre-requisite to integration of bone and survival in fracture repair^61^,^62^. To explore the angiogenic potential of the Mg-Cu alloys, endothelial cell migration, tube formation in tridimensional structures, aortic ring model of angiogenesis, typical gene, and protein expressions in the extracts of Mg-Cu alloys are investigated. Endothelial cell migration follows a series of events in which the endothelial cells extend, contract, throw their rear towards the front, and progress forward^63^. Our results show outstanding cell migration on Mg-0.03Cu, followed by pure Mg and Mg-0.19Cu (Fig. 12). In order to simulate capillary generation in the human body including endothelial cell proliferation and structural form of the capillary network, three-dimensional tubular formation on the Mg-Cu alloys and pure Mg is examined^64^. Nodes, circles, and tubers are orderly on the different samples with incubation time representing the primary, interim and later phases, respectively^65^. Compared to the control, angiogenesis on the Mg-Cu alloys and pure Mg appears earlier and apoptosis begins later. The *in vitro* rat aortic ring model is used to study the outgrowth of microvessels from a three-dimensional tissue fragment into a fibrin matrix to maintain the tissue architecture (Fig. 14). This assay is critical to the understanding of the interactions between the vascular element and supporting tissues^66^. In this study, all the Mg-Cu alloys show angiogenic sprouting into the three-dimensional fibrin matrix. In particular, the microvessel networks generated on Mg-0.03Cu are more extensive, revealing that Mg-0.03Cu can effectively induce the vascular formation during new bone regeneration due to up-regulation of the angiogenesis-related gene and protein expressions (Fig. 15).

Previous studies have demonstrated the vital role of magnesium in modulating microvascular functions^67^. Extracellular magnesium ions are recognized as a receptor-mediated chemoattractant for endothelial cells. Magnesium deficiency may inhibit endothelial cell migration and proliferation, presumably by interfering with some signal transduction pathways triggered by angiogenic factors^68^,^69^,^70^. Meanwhile, Cu has been reported to stimulate the proliferation of endothelial cells in a dose dependent manner *in vitro*^36^ and the ability of Cu to promote wound healing in rats has been linked to the up-regulation of VEGF expressed by the stimulated cells^37^,^71^. Accordingly it is believed that the bioactive Mg and Cu ions released from the Mg-Cu alloys stimulate bone vasculature formation and the angiogenesis mechanism is postulated in Fig. 16(f). The endothelial receptor tyrosine kinase TIE-1, fibroblast growth factor receptor FGFR, and activin receptor-like kinase ACVRL1 are transmembrane receptor proteins which can control the stability of established vessels^72^, stimulate endothelial cells to secrete proteases, and plasminogen activators^73^ and are involved in the TGF-β signaling pathway to regulate vessel maturation in angiogenesis^74^, respectively. The extracellular Mg and Cu may directly activate endothelial receptors on the plasma membranes or indirectly stimulate angiogenesis by inducing the release of angiogenic factors from other cell types. The activated receptors stimulate a number of intracellular signaling pathways, notably the PI3K (phosphoinositide 3-kinase)/Akt which is associated with up-regulation of the apoptosis inhibitor, survivin in endothelial cells and protection of endothelium from death-inducing stimuli^75^.

It has been shown that angiogenesis and osteogenesis are closely linked^61^,^76^. Active vascularization at the injured site is required during the initial step of the bone repair and inadequate blood supply is major contributor to fracture nonunion. Inactivated bone grafts have relatively low angiogenic potential and long-term difficulties such as disintegration and limited resorbable graft size. Similarly, interrelated blood vessel damage is associated with poor bone graft incorporation and fracture healing^77^. Based on our present results, some possible associations exist on the genetic and protein levels coupling the osteogenic and angiogenic processes. Both the bone morphogenetic proteins (Bmps) and activin receptor-like kinase (ACVRL) participate in the transforming growth factor-beta (TGF-β) signaling pathway. Among member of the Bmp family, Bmp4, is known to bind to ACVRL3 and/or ACVRL6 to induce osteoblast differentiation^78^. Bmp9 is a physiological ACVRL1 ligand that plays an important role in the regulation of angiogenesis^79^ and Bmp2 is known to promote vascularization in addition to inducing bone formation^65^,^80^. The Smad protein signal in the downstream of ACVRL1 and BmpR involves the activation and complex interaction in the angiogenic and osteogenic pathways^78^,^81^. Our experimental evidence indicates mutually beneficial coexistence between osteogenesis and angiogenesis when Mg and Cu are release during degradation of Mg-Cu alloys.

## Conclusion

Biodegradable Mg-Cu alloys with multiple functions are designed, fabricated, and investigated. Proper alloying with Cu enhances the mechanical properties of Mg, accelerates the formation of an alkaline environment, and releases bioactive Mg and Cu to synergistically produce long-lasting antibacterial effects. No cytotoxicity towards HUVECs and MC3T3-E1 cells is observed from the alloys and proper release of Mg and Cu during natural degradation of Mg-Cu provides favorable stimulation to osteogenesis and angiogenesis suggesting that the alloys have large potential in orthopedic applications.

## Materials and Experiments

### Materials preparation

The binary Mg-xCu (x = 0.05, 0.2, 0.5 wt.%) alloys were fabricated by melting pure magnesium (99.99%) and pure copper powders (99.9%) in a high purity graphite crucible under SF_6_ (1 vol.%) and CO_2_ (balance). After heating to 750 °C for 40 min, the melt was poured into a steel mold preheated to 300 °C. The compositions of the Mg-xCu ingots determined by inductively-coupled plasma atomic emission spectrometry (ICP-AES, Optima 7300DV, USA) are listed in Table 1. The cast ingots were machined into samples with dimensions of Φ10 × 3 mm^3^, ground with SiC papers up to 2000 grit, ultrasonically cleaned in acetone, absolute ethanol, and distilled water sequentially, and finally dried.

### Microstructure and mechanical properties

The microstructure was examined by optical microscopy (Leica MEF4A, Austria) and scanning electron microscopy (SEM, HITACHI S-3400 N, Japan equipped with energy-dispersive spectrometry, EDS, Oxford INCA energy 300, UK) was employed to determine the secondary phase in the Mg-Cu alloys. The Vicker’s hardness (HV) was measured on a standard micro-hardness tester (TESTOR 1070, Germany) and the tension and compression tests were performed on a standard mechanical testing machine (Zwick Z05, Germany). Five samples from each group were evaluated.

### Degradability

Immersion tests were performed in Hank’s solution with the ratio of sample surface area/extraction medium of 1.25 cm^2^/ml at 37 ± 0.5 °C for 7 days. The solution was refreshed every day and the *pH* was measured at intervals. After immersion for 3 and 7 days, the samples were rinsed with distilled water, dried in air, and photographed by a digital camera (Nikon 3200D, Japan). The corrosion products on the sample surfaces were removed by immersing the samples in chromic acid and the corrosion rate was calculated according to the following equation:


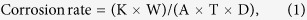


where K is 8.76 × 10^4^, W is the weight loss (g), A is the sample area exposed to the solution (cm^2^), T is the exposure time (h), and D is the density of the material (g cm^−3^). The amounts of Mg and Cu leached from the Mg-Cu alloys to the supernatants were determined by atomic absorption (AAS, Hitachi Z2000, Japan) with the Hank’s solution as the control. The tests were performed in triplicate.

### Antimicrobial assay

The antimicrobial activity of the pure Mg and Mg-Cu alloys was investigated by the bacterial counting method using *Staphylococcus aureus* (*S. aureus*, ATCC 25923) and 304 stainless steel as a positive control. After sterilization by ultraviolet irradiation, the sterilized Hank’s solution was introduced onto the sample at a density of 1.25 cm^2^/ml and kept at 37 ± 0.5 °C for 3, 6, 12, 24, 72, and 120 h. Afterwards, the solutions were withdrawn. Part of the solution was adjusted to a pH of 7.4 with HCl and the other part was unchangeable. The bacterial suspensions with a concentration of (1–10) × 10^6^ cfu/mL of *S. aureus* were introduced to the solutions at volume ratio of 1:9 and the samples were incubated at 37 °C for 24 h. The co-cultured bacterial suspensions were diluted to (1–10) × 10^3^ cfu/mL. 0.1 mL of the diluted bacterial suspension was added to nutrition agar plates and spread evenly, followed by further incubation again at 37 °C for 24 h before counting the bacteria colonies.

### Cell culture

Murine calvarial preosteoblasts (MC3T3-E1, ATCC CRL-2594) and human umbilical vein endothelial cells (HUVECs, ATCC CRL-1730) were employed to examine the cytocompatibility of the Mg-Cu alloys. They were cultured in the modified Eagle’s medium alpha (α-MEM, Hyclone, USA) and endothelial cell medium (Sciencell, USA), respectively, with 10% fetal bovine serum (FBS, Corning, USA ) at 37 °C in a humidified atmosphere of 5% CO_2_. The culture medium was changed every three days.

### Preparation of extracts

Extracts were prepared using serum-free α-MEM and serum-free endothelial cell medium as the extraction medium with a ratio of extraction medium/sample surface area of about 1.25 cm^2^/ml in a humidified atmosphere containing 5% CO_2_ at 37 °C for 24 h according to the International Standard Organization (ISO 10993-5). Prior to immersion, the samples were sterilized by ultraviolent irradiation for 30 min and after immersion, the supernatant was withdrawn, centrifuged, and filtered to prepare the extraction medium. It was refrigerated at 4 °C before cell tests and the extract from pure Mg served as the control.

### Cytotoxicity and cell proliferation

The cytotoxicity and cell proliferation were evaluated by an indirect contact assay according to ISO 10993-5. MC3T3-E1 and HUVECs cells were seeded on 96-well cell culture plates at a density of 5 × 10^3^ cells/100 mL and incubated for 24 h to allow cell attachment. The medium was then replaced with 100 μL of the extracts. After culturing for 1, 3, and 5 days, MTT solutions were added to each well and incubated at 37 °C for 4 h to form formazen which was then dissolved using dimethyl sulfoxide (DMSO). The optical density (OD) was determined on a microplate reader (Thermo Scientific Multiskan GO, USA) at 490 nm with a reference wavelength of 570 nm to determine the cell viability in comparison with the control.

### Cell morphology

The MC3T3-E1 and HUVECs cells were seeded on 12-well cell culture plates at a density of 2 × 10^4^ cells/well for 24 h to allow cell attachment. The medium except the control group was then replaced by 500 μL of the extracts. After incubation for 12 h, the unattached cells were removed by rinsing with phosphate buffered saline (PBS) solution. The cells were fixed with 4% paraformaldehyde at 4 °C for 30 min and permeabilized with 0.5% Triton X-100 in PBS solution for 5 min. Afterwards, the cells were rinsed with PBS solution and the F-actin stress fibers and nuclei were stained with rhodamine phalloidin (Cytoskeleton, USA) and 40, 6-diamidino-2- phenylindole (DAPI, Dojindo, Japan), respectively. The cytoskeleton and cell nuclei were examined by fluorescence microscopy (Olympus IX71, Japan).

### Alkaline phosphatase (ALP) activity

The MC3T3-E1 cells were plated on 12-well cell culture plates at a density of 3 × 10^4^ cells/well and cultured in different extracts supplemented with 100 nM dexamethasone, 0.2 mM ascorbic acid, and 10 mM β-glycerophosphate. The extracts were changed every 3 days. After 4, 7 and 14 days, the cells were washed three times with PBS and lysed in 0.2 vol.% Triton X-100. The alkaline phosphatase (ALP) activity was determined by a colorimetric assay using an ALP reagent containing p-nitrophenyl phosphate (p-NPP) as the substrate (Beyotime, China). The absorbance of p-nitrophenol was monitored at 405 nm. The intracellular total protein content was determined using the MicroBCA protein assay kit (Thermo Pierce, USA) and the ALP activity was normalized to the total protein content.

### Extracellular matrix (ECM) mineralization and collagen secretion

ECM mineralization and collagen secretion by the MC3T3-E1 cells in the Mg-Cu alloys extracts were assessed by the Alizarin Red and Sirius Red staining, respectively. After culturing for 15 days, the cells with an initial concentration of 3 × 10^4^/well were washed and fixed. Afterwards, they were stained using 40 mM Alizarin Red (Sigma) at a pH of 4.2 to show mineralization or 0.1% Sirius Red (Sigma) to reveal the collagen. The unbound stain was washed with distilled water or 0.1 M acetic acid prior to photographing by a digital camera (Nikon D3200, Japan). In the quantitative analysis, the Alizarin Red or Sirius Red stain was dissolved in 10% cetylpyridinum chloride in 10 mM sodium phosphate (pH 7) or 0.2 M NaOH/methanol (1:1). The absorbance was measured at 620 nm or 540 nm.

### Cell migration

A wound-healing assay was used to evaluate the migration of HUVECs in the extracts of pure Mg and Mg-Cu alloys. Cells at a density of 5 × 10^4^ cells/well were cultured on the 12-well cell culture plates for 24 h to reach confluency. The monolayers were wounded with a plastic pipette at a width of approximately 250 μm. After washing with PBS, the cells were incubated for another 6 and 12 hours and observed by inverted phase microscopy (Olympus IX71, Japan).

### *In vitro* angiogenesis

An *in vitro* angiogenesis assay was conducted using the Matrigel™ basement membrane matrix (Corning, 356234) prepared for 3D cell culturing. The 96-well tissue culture plates were coated with the Matrigel™ matrix on ice using cooled pipets and placed on plates at 37 °C for 30 minutes. The HUVECs (3 × 10^4^ cells/well) were incubated in different material extracts with 1% FBS on Matrigel for 4, 8, and 16 hours at 37 °C. At each time point, the cells were photographed from five random fields on an inverted phase microscope (Olympus IX71, Japan).

### Aortic ring model of angiogenesis

The experimental protocol was approved by the Ethics Review Committee for Animal Experimentation of Shenzhen Institute of Advanced Technology, Chinese Academy of Sciences, Shenzhen, China (No. SIAT-IRB-140110-YYS-WLEI-A0010). This experiment was carried out in accordance with the approved guidelines. Three male Sprague Dawley (SD) rats were obtained from the Animal Test Center of Shenzhen Institute of Advanced Technology, Chinese Academy of Sciences. After the SD rats were sacrificed and dissected, the fresh thoracic aortas were processed immediately after harvesting. The thoracic aortas were cut into aortic rings 2 mm in diameter and 1 mm thick and immediately embedded into rat tail collagen gels. The rat tail collagen gels (Sigma, USA) were pre-prepared on 48-well plates using an ECM serum free medium. After gelation of the aortic rings in gels at 37 °C for 30 min, the extracts of the Mg-Cu alloys and pure Mg were added to each well and the endothelial cell medium serum free medium was taken as the control. The plates were kept in a humidified atmosphere with 5% CO_2_ at 37 °C for 7 and 14 days and the individual well containing the rat thoracic aorta rings was examined by optical microscopy (Olympus IX71, Japan).

### Real-time polymerase chain reaction (RT-PCR)

RT-PCR was used to evaluate the expression of the osteogenesis-related and angiogenesis-related genes. The MC3T3-E1 and HUVECs cells were seeded on 12-well cell culture plates at a cell density of 3 × 10^4^cells/well and after 24 h, the culture medium was replaced by different material extracts supplemented with fresh serum. The MC3T3-E1 cells were cultured for 7, 10 and 14 days and the HUVECs were cultured for 3 days. The total RNA was isolated using a Trizol reagent (Invitrogen, USA) and the concentration of RNA was determined by monitoring the optical absorbance at 260 nm on the Thermo 2000c (USA). 1 μg of the RNA was reversely transcribed into complementary DNA (cDNA) using Superscript III (Invitrogen, USA) in a volume of 20 μl. The forward and reverse primers of the selected genes are listed in Table 1S in the supporting information. The expressions of the osteogenesis-related genes, including runt-related transcription factor 2 (Runx2), bone morphogeneticprotein-2 (Bmp2), bone sialoprotein (Bsp), and Type I collagen alpha 1 (Cola1) as well as expressions of the angiogenesis-related genes including activin A receptor type II-like 1 (ACVRL1), endothelialnitric oxide synthase (eNOs), tyrosine kinase with immunoglobulin-like and EGF-like domains 1 (TIE-1), and fibroblast growth factor receptor 1 (FGFR1) were quantified by Real-time PCR (Biorad CFX96, USA) on the SYBR Green PCR Master Mix (Applied Biosystems, USA). The relative mRNA expression level of each gene was normalized to the housekeeping gene β-actin (mouse β-actin for osteogenesis-related genes expression and human β-actin for angiogenesis-related genes expression) and determined by the Ct values.

### Western blot analysis

Western blot analysis was used to evaluate the expressions of osteogenesis and angiogenesis-related proteins. The cultured cells (MC3T3-E1 cells and HUVECs) were harvested in a lysis buffer (Beyotime, China) and the protein concentrations were measured using a BCA reagent (Thermo Scientific, USA). The total protein (50 μg) was separated using the SDS-PAGE gel and transferred to a PVDF membrane (Merck Millipore, USA). The membrane was incubated overnight at 4 °C with anti-Runx2 (Cell Signaling Technology), anti-Bmp2 (Santa Cruz Biotechnology, USA), anti-ACVRL1 (Santa Cruz Biotechnology, USA), and anti-TIE1 (Merck Millipore, USA). The bound primary anti-bodies were recognized by HRP-linked secondary antibodies (Santa Cruz Biotechnology, USA). The protein bands were visualized using the enhanced chemiluminescence substrate kit (Merck Millipore, USA).

### Statistical analysis

The cell tests were performed in triplicate. The experimental results were expressed as mean ± standard deviations and the data were analyzed using SPSS 13.0 software. A paired t-test (Student’s t-test) was performed with *p* < 0.05 considered to be statistically significant and *p* < 0.01 highly statistically significant.

## Additional Information

**How to cite this article**: Liu, C. *et al.* Biodegradable Mg-Cu alloys with enhanced osteogenesis, angiogenesis, and long-lasting antibacterial effects. *Sci. Rep.* **6**, 27374; doi: 10.1038/srep27374 (2016).

## Supplementary Material

Supplementary Information

## Figures and Tables

**Table 1:** Chemical compositions of Mg-Cu alloys (wt%).

Nominal composition		Mg-0.05Cu	Mg-0.2Cu	Mg-0.5Cu
Actual composition	Cu	0.03	0.19	0.57
	Mg	Balance		
