# Supplementary material for: Biodegradable Mg-Cu alloys with enhanced osteogenesis, angiogenesis, and long-lasting antibacterial effects
Source: Sci Rep. 2016 Jun 7;6:27374. doi: 10.1038/srep27374 (PMC4895436; doi:10.1038/srep27374)
Supplement: Supplementary Information [file srep27374-s1.doc]

**Supplementary Material**

**Biodegradable Mg-Cu alloys with enhanced osteogenesis, angiogenesis, and long-lasting antibacterial effects**

Chen Liu^1,2,3,#^, Xuekun Fu^3,#^, Haobo Pan^3^, Peng Wan^1^, Lei Wang^3^, Lili Tan^1^, Kehong Wang^2^, Ying Zhao^3,*^, Ke Yang^1,*^, Paul K. Chu^4^

^1^ Institute of Metal Research, Chinese Academy of Sciences, Shenyang, China

^2^ Department of Materials Science and Engineering, Nanjing University of Science and Technology, Nanjing, China

^3^ Shenzhen Institutes of Advanced Technology, Chinese Academy of Sciences, Shenzhen, China

^4^ Department of Physics and Materials Science, City University of Hong Kong, Tat Chee Avenue, Hong Kong, China

* Corresponding authors

Tel.: +86 0755 86585229. E-mail address: ying.zhao@siat.ac.cn (Y. Zhao).

Tel.: +86 024 23971628. E-mail address: kyang@imr.ac.cn (K. Yang).

# These two authors contributed equally to this paper.


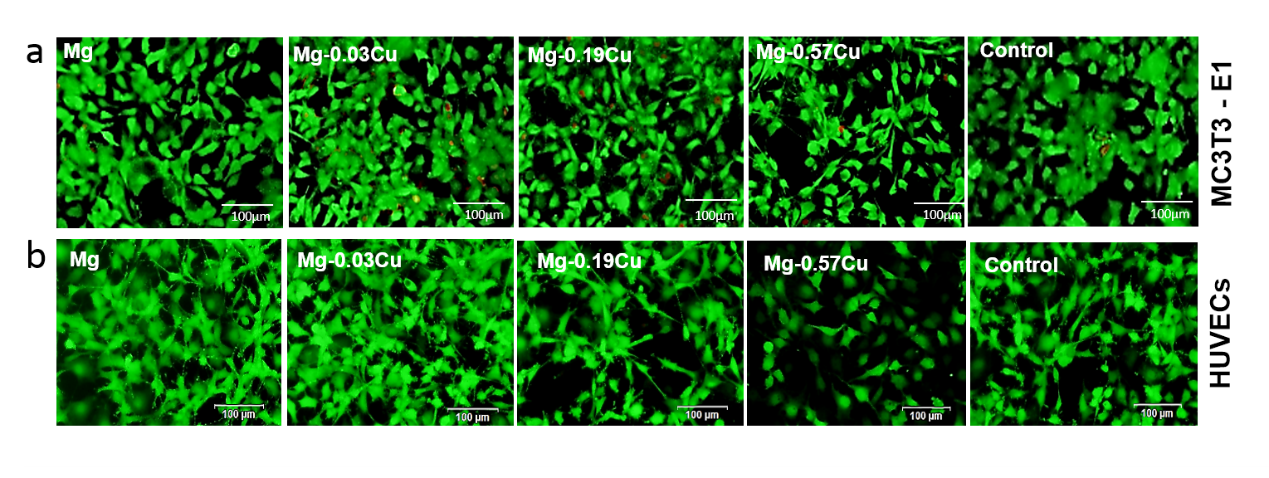


**Fig.1S** Live/dead staining of MC3T3-E1 cells (a) and HUVECs (b) after 24h of incubation in the different extracts. Calcein-AM for live cells (green) and iodide pyridine for dead cells (red).

**Table 1S** Primer pairs used in real-time PCR analysis.

| Gene | Forward primer | Reverse primer |
| --- | --- | --- |
| mouse β-actin  human β-actin  Runx2  Bmp2  Bsp  Cola1  ACVRL1  eNOs  TIE-1  FGFR1 | 5'-GGCTGTATTCCCCTCCATCG-3'  5'-CATGTACGTTGCTATCCAGGC-3'  5'-AGAGTCAGATTACAGATCCCAGG-3'  5'-GGGACCCGCTGTCTTCTAGT-3'  5'-CAGGGAGGCAGTGACTCTTC-3'  5'-GCTCCTCTTAGGGGCCACT-3'  5'-CATCGCCTCAGACATGACCTC-3'  5'-TGATGGCGAAGCGAGTGAAG-3'  5'-AAGCAGACAGACGTGATCTGG-3'  5'-CCCGTAGCTCCATATTGGACA-3' | 5'-CCAGTTGGTAACAATGCCATGT-3'  5'-CTCCTTAATGTCACGCACGAT-3'  5'-TGGCTCTTCTTACTGAGAGAGG -3'  5'-TCAACTCAAATTCGCTGAGGAC-3'  5'-AGTGTGGAAAGTGTGGCGTT-3'  5'-CCACGTCTCACCATTGGGG-3'  5'-GTTTGCCCTGTGTACCGAAGA-3'  5'-ACTCATCCATACACAGGACCC-3'  5'-GCACGATGAGCCGAAAGAAG-3'  5'-TTTGCCATTTTTCAACCAGCG-3' |
